# Long-Term Follow-Up of a Patient with a Novel Homozygous *ASTN1* Variant: A Case Report

**DOI:** 10.3390/neurolint18040072

**Published:** 2026-04-19

**Authors:** Buşra Kasap, Dilek Uludağ Alkaya, Nilay Güneş, Salih Türk, Barış Korkmaz, Beyhan Tüysüz

**Affiliations:** 1 Department of Pediatric Genetics, Cerrahpaşa Medical Faculty, Istanbul University-Cerrahpaşa, Istanbul 34098, Turkey; busrakasap@ogr.iu.edu.tr (B.K.); dilek.uludagalkaya@iuc.edu.tr (D.U.A.); nilay.gunes@iuc.edu.tr (N.G.); salih.turk@iuc.edu.tr (S.T.); 2 Institute of Health Sciences, Istanbul University, Istanbul 34126, Turkey; 3 Department of Neurology, Cerrahpaşa Medical Faculty, Istanbul University-Cerrahpaşa, Istanbul 34098, Turkey; bkorkmaz@iuc.edu.tr; 4 Department of Pediatrics, Istanbul Atlas University, Istanbul 34408, Turkey

**Keywords:** *ASTN1*, neurodevelopmental disorder, exome sequencing, microcephaly

## Abstract

Background/Objectives: Severe neurodevelopmental disorders caused by homozygous *ASTN1* variants have recently been reported. The aim of this study is to present the expanded phenotype and prognostic findings through a longitudinal follow-up of a patient with a homozygous *ASTN1* variant. Methods: We conducted a 15-year clinical evaluation of a girl who initially presented at 10 years of age. The genetic etiology was investigated using exome sequencing. Results: The patient had a profound intellectual disability, severe expressive language delay, and infantile-onset epilepsy. She also had microcephaly, achieved independent walking at age 7 and had speech limited to only two words at admission. A novel homozygous frameshift variant, c.2096del (p.Cys699Serfs*22), in *ASTN1* was identified. Over the follow-up period, her postnatal microcephaly became more pronounced, and she experienced a late relapse into generalized tonic–clonic seizures after a decade-long remission. She remains entirely dependent on caregivers for basic self-care at age 25. Conclusions: *ASTN1*-related phenotype is associated with a severe neurodevelopmental disease, and the late relapse of seizures after prolonged remission highlights the need for lifelong neurological monitoring and multidisciplinary care.

## 1. Introduction

The developing nervous system is shaped by coordinated interactions among neurons, glial cells, and the extracellular matrix, which collectively regulate the highly ordered process of neuronal migration. Among the molecules orchestrating these interactions, astrotactin proteins serve as key mediators of neuron–glia communication [1,2]. In particular, *ASTN1* encodes astrotactin-1, a neuronal adhesion molecule that bridges neuron–glia adhesion through asymmetric interactions with N-cadherin (CDH2), a classical adhesion molecule linking the plasma membrane to the actin cytoskeleton and regulating intercellular junctions during neural development. By engaging in cis interactions with neuronal CDH2 and trans interactions with glial CDH2, ASTN1 stabilizes the migration junction and enables glial-guided neuronal migration [3]. Experimental studies in *ASTN1* knockout mice have shown that loss of ASTN1 impairs glia-guided neuronal migration due to reduced neuron–glia adhesion. Mutant neurons fail to maintain contact with radial glial fibers, resulting in delayed granule cell migration, and disrupted cerebellar architecture. These structural abnormalities are associated with reduced cerebellar size and impaired motor coordination, supporting a loss-of-function mechanism [4].

The role of astrotactins in human disease has gained increasing recognition over the past decade. Initially, large cohort studies between 2015 and 2020 proposed *ASTN1* as a candidate gene for brain malformations, epilepsy, and intellectual disability (ID) [5,6,7,8,9]. Ayaz et al. became the first to directly link an *ASTN1* variant to a distinct neurodevelopmental phenotype in a detailed case report [10]. Most recently, a multicenter study by Levine et al. definitively established bi-allelic *ASTN1* variants as the cause of a diverse neurodevelopmental disorder in a cohort of 18 patients [11].

The long-term natural history of *ASTN1*-related disorder remains poorly delineated. Here, we present a patient with a novel homozygous *ASTN1* frameshift variant who has been followed longitudinally for 15 years.

## 2. Detailed Case Description

**Figure 1:**
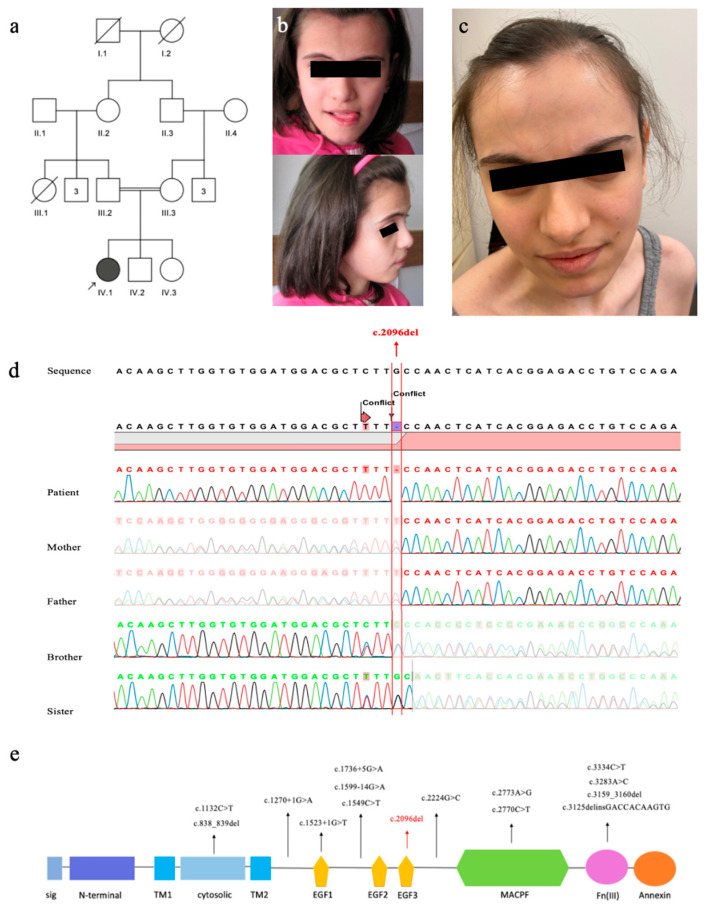
Pedigree of the family; the arrow indicates the index patient (IV:1) (**a**), The photographs of patient at the age of 10 (**b**) and 25 (**c**): Note broad forehead and triangular face. Sanger electropherogram confirming the c.2096del (p.Cys699Serfs*22) variant (**d**): The patient is homozygous, while both parents and unaffected siblings are heterozygous for this variant. Schematic representation of the ASTN1 protein domain architecture and the distribution of disease-causing variants (**e**): Structural modules include the signal peptide (sig), epidermal growth factor-like domains (EGF1–3), membrane attack complex/perforin (MACPF) domain, fibronectin type III (FNIII) domain, annexin-like domain, and transmembrane regions (TM1–2). The homozygous variant from the current study (red color) is highlighted. The previously reported homozygous variants are shown in black.

A 10-year-8-month-old girl, born to first-cousin parents (Figure 1a), was admitted for evaluation of severe ID and epilepsy. She was delivered at term with a birth weight of 2650 g (−1.88 SDS), length of 50 cm (−0.39 SDS), and head circumference (HC) of 34 cm (−0.7 SDS). Developmental milestones were markedly delayed: head control was achieved at 8 months, unsupported sitting at 3 years, and independent walking at 7 years. Speech development was severely impaired, with first recognizable words emerging at 4 years of age.

At her initial examination at age 10.7 years, weight was 25 kg (−1.92 SDS), height 129 cm (−2.08 SDS), and HC 50 cm (−2.15 SDS). Dysmorphic features included a broad forehead, triangular face (Figure 1b), long columella, highly arched palate, and hypermobile fingers. She could say only two words.

At her last examination at age 25 (Figure 1c), her weight was 44.4 kg (−2.53 SDS), height was 159 cm (−0.7 SDS), and HC was 52 cm (−3.32 SDS), consistent with profound microcephaly (Table 1). Expressive language remained severely restricted to a vocabulary of approximately 20 words. She was able to walk independently but with gait instability and frequent falls, could manage stairs and feeding only with support, and was unable to dress or undress independently.

Seizures manifested at 1 year of age and were initially controlled with sodium valproate. After a period of remission, generalized tonic–clonic (GTC) seizures relapsed at 13 years, requiring carbamazepine treatment. At that time, EEG demonstrated generalized multifocal spike–wave discharges, while brain MRI revealed two non-specific nodular hyperintense lesions (up to 3 mm) in the subcortical white matter of the left frontal lobe. Carbamazepine treatment was still ongoing at 25 years of age.

Exome sequencing identified a novel homozygous *ASTN1* (NM_004319.3):c.2096del (p.Cys699Serfs*22) variant. The variant was confirmed by Sanger sequencing, with both parents and unaffected siblings shown to be heterozygous carriers (Figure 1d). This variant is absent from population databases (gnomAD v4.0: 0) and is classified as likely pathogenic based on ACMG criteria (PVS1, PM2). All previously reported pathogenic variants, together with our novel variant, were mapped onto the protein’s modular architecture using data from the UniProt and Simple Modular Architecture Research Tool databases (Figure 1e).

**Figure 2:**
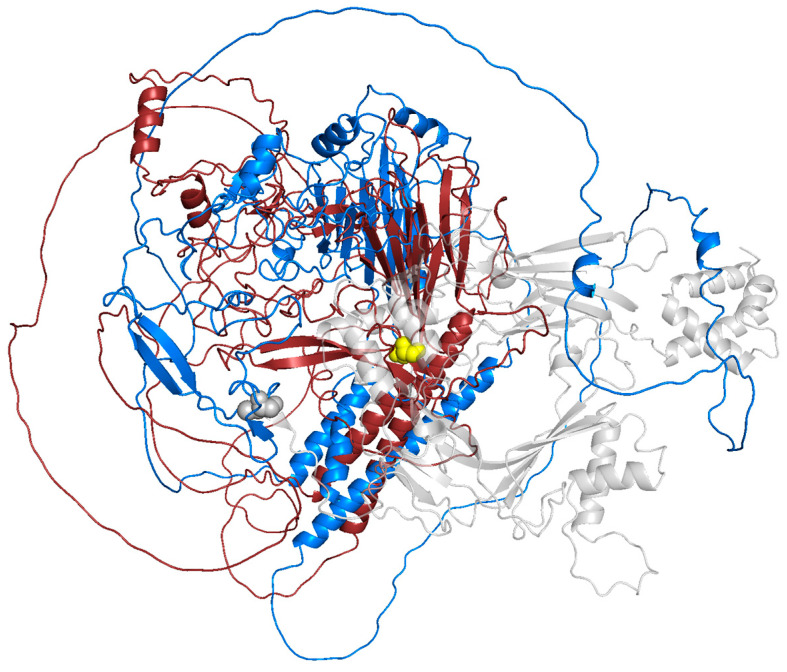
Structural superimposition of wild-type and mutant ASTN1 proteins. The structures are superimposed to compare their conformational states. From the N-terminus to residue 699, the wild-type (marine blue) and mutant (ruby red) proteins are overlapped, showing conserved structural domains until the mutation site. The yellow spheres indicate the initiation point of the p.Cys699Serfs*22 frameshift. The transparent gray region highlights the C-terminal loss (residues 699–1310) that is present in the wild-type but absent in the truncated mutant.

To assess the structural impact of the variant on the ASTN1 protein, a comparative 3D modeling analysis was performed (Figure 2). The wild-type ASTN1 structure was obtained from the AlphaFold Protein Structure Database (UniProt ID: O14525, Model ID: AF-O14525-F1). The mutant protein structure, consisting of the first 698 wild-type residues followed by a 21-residue frameshift tail, was modeled using the ColabFold/AlphaFold2, based on the truncated amino acid sequence [12,13]. Structural superimposition of the wild-type and mutant models was performed and visualized using PyMOL (v2.5).

The Root Mean Square Deviation (RMSD) was calculated for the conserved N-terminal region to ensure alignment accuracy. A high RMSD value was observed when comparing the full-length structures (26.415 Å), primarily reflecting the C-terminal truncation and differences in sequence length rather than local structural changes in the conserved region.

**Figure 3:**
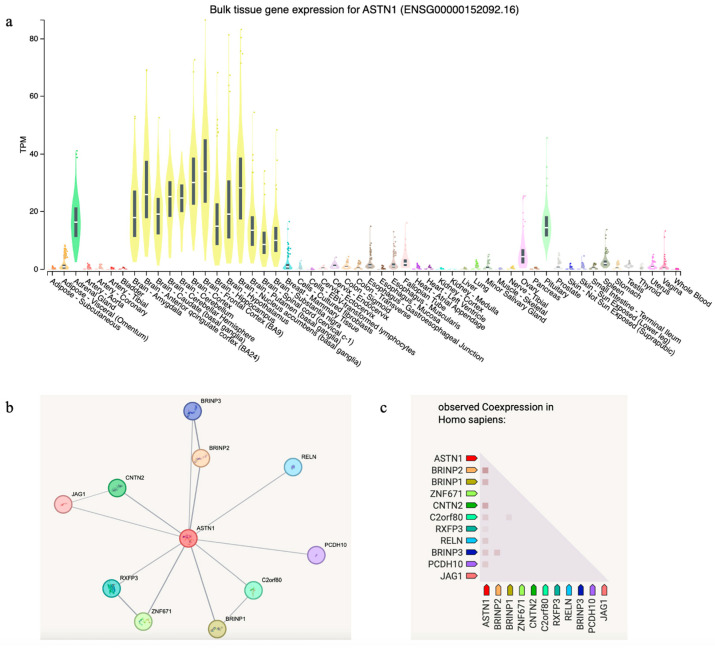
Spatial and temporal expression analysis of *ASTN1*. (**a**) Violin plot of *ASTN1* expression across normal human tissues from the GTEx Portal; data is color-coded by organ system to facilitate tissue-specific comparison (https://www.gtexportal.org/home/gene/ASTN1, access date: 16 April 2026). (**b**) Protein–protein interaction network centered on *ASTN1* and its functional partners as identified by the STRING database. (**c**) Co-expression heatmap showing the correlation between *ASTN1* and the genes identified in the interaction network.

To explore the expression profile and molecular interactome of *ASTN1*, we conducted an *in silico* gene expression analysis using the GTEx Portal, which confirmed that *ASTN1* is predominantly expressed in human brain tissues, with peak expression levels observed in the cerebellum and cerebral cortex (Figure 3a). Beyond the central nervous system, notable expression levels were identified in the adrenal gland, pituitary, and minor salivary glands. Furthermore, we performed a functional protein–protein interaction analysis using the STRING database (v12.0), which revealed a robust network in which *ASTN1* showed significant co-expression and interaction scores with several key neurodevelopmental genes, most notably *CNTN2* and *BRINP2* (Figure 3b,c).

## 3. Discussion

In this study, a novel frameshift *ASTN1* variant was described in a patient with microcephaly, severe ID, and epilepsy. Although this disease is newly described, a recent large cohort study has definitively identified *ASTN1* as a disease-causing gene [11]. Our study confirms the pathogenicity of *ASTN1* and provides crucial long-term follow-up of an adult patient.

The mutation spectra of *ASTN1* in reported patients include missense, nonsense, frameshift, and splice-site variants [11]. As illustrated in Figure 1e, the spatial distribution of these mutations reveals the absence of a distinct mutational hotspot; instead, they are widely dispersed across the entire extracellular landscape of the protein. *In silico* structural modeling revealed that the p.Cys699Serfs*22 variant leads to a massive C-terminal truncation, effectively removing 591 amino acids from the ASTN1 protein (Figure 2). This loss encompasses the entire MACPF domain (residues 714–955) and the FNIII domain (residues 1021–1110), which are critical for ASTN1-mediated glial-guided neuronal migration, given that ASTN1 interacts directly with CDH2 through its extracellular C-terminal domains [3].

*ASTN1* is predominantly expressed in the human brain, with highest levels in the cerebellum and cerebral cortex (Figure 3a). Co-expression analysis revealed moderate associations between *ASTN1* and several neurodevelopmental genes, with the highest score observed for *BRINP2*, followed by *CNTN2* (Figure 3c). *BRINP2* acts as a critical cell-cycle inhibitor facilitating terminal neuronal maturation, and *CNTN2* organizes the molecular scaffolds necessary for action potential stability. Overall, these findings support the involvement of *ASTN1* in a coordinated network regulating neuronal migration, adhesion, and cortical development.

Microcephaly has previously been reported in only two out of ten patients (Table 1). In contrast, although her head circumference was within the normal range at birth (−0.7 SDS), our patient demonstrated progressive postnatal deceleration, reaching −2.15 SDS at 10.8 years and −3.32 SDS by 25 years of age, suggesting that microcephaly may represent an additional feature associated with *ASTN1* deficiency. Given the established role of *ASTN1* in glia-guided neuronal migration, its deficiency may lead to delayed neuronal migration and increased apoptosis, which may contribute to impaired brain growth [4]. However, given the limited number of reported cases, this interpretation should be considered with caution, and further studies are needed to clarify the relationship between *ASTN1* variants and microcephaly.

Dysmorphic facial features of our patient were subtle, including a broad forehead, triangular face, long columella, and highly arched palate. Similarly non-specific dysmorphic features were reported in the majority of the Levine et al. [11] cohort (11 of 18 individuals), although a recognizable facial gestalt was not established. In their cohort, specific features such as frontal bossing, micrognathia, hypertelorism, and upslanting palpebral fissures were detailed in only one individual [11].

The neurodevelopmental profile of our patient, characterized by severe ID and profound expressive language delay, aligns with the severe end of the phenotypic spectrum recently established by Levine et al. [11]. In their cohort of 18 individuals (average age = 5.5 years, range: 11 months–12 years), 39% (7/18) remained non-verbal, a feature mirrored by our 25-year-old patient who possesses no functional speech. Regarding motor development, nearly half of the reported cohort (8 of 18) were non-ambulatory at the time of their last evaluation. Given the relatively young median age of this group, our patient’s longitudinal clinical course, characterized by severe speech delay and gait instability, provides valuable insight into long-term motor outcomes.

The reported cranial imaging findings are variable, ranging from completely normal brain imaging to severe structural malformations such as lissencephaly and polymicrogyria (Table 1). The most frequently observed structural defects involved the corpus callosum and cerebellar anomalies, while four individuals presented with entirely normal MRIs. In our patient, brain MRI revealed only non-specific nodular hyperintense lesions in the subcortical white matter without gross malformations. This structural heterogeneity suggests that the neuroradiographic consequences of *ASTN1* deficiency lie on a broad clinical spectrum.

The seizures in *ASTN1*-related disorders include infantile spasms, isolated tonic or atonic seizures in infancy, and GTC seizures (occasionally triggered by fever), often manifesting as drug-resistant epilepsy requiring multiple antiseizure medications [10]. Consistent with this, our patient presented with infantile-onset seizures. However, our patient had a late relapse after a remission of over a decade. Although studies directly linking ASTN1-mediated neuron–glia interactions to epilepsy pathophysiology are limited, impaired neuronal migration and disrupted neuron–glia adhesion may contribute to abnormal network formation and increased excitability underlying epileptic seizures [14].

## 4. Conclusions

In conclusion, this case report expands the clinical spectrum of *ASTN1*-related neurodevelopmental disorder by describing a novel homozygous frameshift variant, documenting postnatal microcephaly, and providing long-term follow-up data into adulthood. Notably, the recurrence of seizures after a decade-long remission highlights the need for continuous neurological surveillance in adult patients.

## Figures and Tables

**Table 1:** Comparison of clinical and neuroradiological features of reported patients with *ASTN1* variants and the patient presented here.

	Karaca et al., 2015 [5]. Levine et al., 2026 [11]	Anazi et al., 2016 [6], Levine et al., 2026 [11]	Wiszniewski et al., 2018 [7], Levine et al., 2026 [11]	Maddirevula et al., 2020 [8], Levine et al., 2026 [11]	Mitani et al., 2021 [9], Levine et al., 2026 [11]	Ayaz et al., 2025 [10]	Levine et al., 2026 [11]	This Study
**Number of families**	1	1	1	1	1	1	6	1
**Number of patients**	2	4	1	2	1	1	7	1
**Gender (F)**	2/2	2/4	0/1	1/2	0/1	1/1	1/7	1/1
**Consanguinity**	1/1	1/1	0/1	1/1	0/1	1/1	5/6	1/1
**Global developmental** **delay**	2/2	4/4	1/1	2/2	1/1	1/1	7/7	1/1
**Microcephaly**	1/1	0/1	NR	NR	NR	0/1	1/7	1/1
**Ataxia**	NR	NR	NR	1/2	0/1	0/1	3/7	1/1
**Seizure/abnormal EEG**	2/2	1/1	1/1	1/2	1/1	1/1	5/7	1/1
**Brain MRI findings**								
Normal	0/2	0/1	0/1	1/2	0/1	0/1	3/7	0/1
Diffuse cerebral atrophy	0/2	0/1	0/1	0/2	0/1	1/1	0/7	0/1
Cortical atrophy	0/2	0/1	0/1	0/2	0/1	0/1	1/7	0/1
Subcortical signal changes	0/2	0/1	0/1	0/2	0/1	0/1	0/7	1/1
Hydrocephalus	0/2	0/1	0/1	0/2	1/1	0/1	0/7	0/1
Cerebellar hypoplasia	0/2	0/1	0/1	1/2	1/1	0/1	3/7	0/1
Thin CC	2/2	0/1	0/1	0/2	1/1	1/1	3/7	0/1
Colpocephaly	0/2	0/1	0/1	0/2	1/1	0/1	0/7	0/1
Abnormal hippocampus	0/2	1/1	0/1	0/2	0/1	0/1	0/7	0/1
Polymicrogyria	0/2	0/1	1/1	0/2	0/1	0/1	0/7	0/1
Lissencephaly	0/2	0/1	0/1	0/2	1/1	0/1	0/7	0/1

Abbreviations: CC Corpus callosum, EEG Electroencephalogram, F Female, NR Not reported.

## Data Availability

The data that support the findings of this study are available from the corresponding author upon request. The data are not publicly available due to privacy and ethical restrictions regarding human genetic data.

## Abbreviations

The following abbreviations are used in this manuscript:

ID	Intellectual disability
GTC	Generalized tonic–clonic
HC	Head circumference
